# Trustworthiness and Ethics in Vaccine Hesitancy: Mind the Gap

**DOI:** 10.1093/phe/phag011

**Published:** 2026-07-13

**Authors:** M van den Hoven

**Affiliations:** Amsterdam University Medical Center, Amsterdam, The Netherlands

## Abstract

We know that vaccine hesitancy is closely related to distrust in health organisations, pharmaceutical industry or governments (Vuolanto *et al.* 2024). At the end of the book, I am left with the question what is necessary to bridge the gap of distrust and how this relates to discussions on coercive measures. Intuitively, it seems necessary to understand the different types of distrust non-compliant parent groups have and Pierik and Verweij discuss these in Chapter 3 in order to offer tailored approaches to build trust. In Chapter 9, Pierik and Verweij discuss the lack of trust more specifically, but I question if their approach is well thought through: they are ambivalent on a so-called information deficit perspective on trust, which they reject and also seem to assume at the same time. I will first look at the various sources of distrust that Pierik and Verweij distinguish before I discuss their view on trust and trustworthiness.

## Introduction

Over the past decade, debates on vaccine hesitance have been reignited by outbreaks of infectious diseases, like measles outbreaks in Disneyland California, UK and in Italy (McCarthy, 2015; Ghebrehewet *et al.*, 2016; Siani, 2019) and also by the COVID-19 pandemic (Verger and Dubé, 2020). The WHO has, already in 2019, put vaccine hesitance in the top ten of health threats (Galagali *et al.*, 2022) and this hesitancy is the starting point in *Inducing Immunity. Justifying immunization policies in times of vaccine hesitance*, written by Roland Pierik and Marcel Verweij (Pierik and Verweij, 2024). The book inquires if coercive measures can be justified in liberal-democratic societies when dealing with deep controversies concerning vaccination (ibid: 16). It is a fair and urgent question, given the decrease of immunisation rates in many countries, which are challenging herd immunity. We know that vaccine hesitancy is closely related to distrust in health organisations, pharmaceutical industry or governments (Vuolanto *et al.* 2024). At the end of the book, I am left with the question what is necessary to bridge the gap of distrust and how this relates to discussions on coercive measures. Intuitively, it seems necessary to understand the different types of distrust non-compliant parent groups have and Pierik and Verweij discuss these in Chapter 3 in order to offer tailored approaches to build trust. In Chapter 9, Pierik and Verweij discuss the lack of trust more specifically, but I question if their approach is well thought through: they are ambivalent on a so-called information deficit perspective on trust, which they reject and also seem to assume at the same time. I will first look at the various sources of distrust that Pierik and Verweij distinguish before I discuss their view on trust and trustworthiness.

## Trust and Parental Hesitance

In Chapter 3, the authors distinguish four groups of people who forgo vaccination offers for their children. These are parents who (i) have conscientious objections for religious reasons (ii) embrace anthroposophist views on health; (iii) have deep concerns about the risks and benefits of vaccination, or (iv) have ‘epistemic concerns’ (ibid: 35–45). The different sources of objections in each of these four groups can also lead to different types (or sources of) distrust. Pierik and Verweij do not discuss trust issues regarding the first two groups, namely those parents who base their refusal on religious objections, like divine providence (ibid: 35–37),^1^ and those that embrace the Steiner anthroposophy perspective, embracing a diverting view on the nature and burden of infectious diseases in life. Interesting to know about the first two groups is that they are easily identifiable, and also their geographical spread in countries is well-known, as the famous ‘Bible belt’ in the Netherlands. It can put these communities themselves more at risk when infectious outbreaks occur.

The other two groups are more diffuse in social and cultural backgrounds, and can be found at all levels of the population and regions. The third group consists both of parents who share certain ideals or views on ‘natural healing’, but also of parents who ‘find support in more down-to-earth sociopolitical sentiments like a lack of trust in government, public health authorities, scientific institutions and big farma’ (ibid: 38). They can believe, for example that the pharmaceutical industry is primarily motivated by the desire ‘to sell as many vaccines as possible’ (ibid: 40). The distrust is fuelled by suspicion. Finally, the group of parents who have epistemic concerns, display ‘individual-oriented concerns’ and seek ‘what is best *for their child*’ while vaccine policies are primarily focusing on the benefits at population level. The result is a critical stance towards science and invites to take other (non-scientific) sources like personal anecdotes also into account and to ‘do their own research’ and ‘make up their own mind’ about the evidence base for vaccines (ibid: 45). Distrust here is more epistemic, in that other knowledge sources and own interpretations are trusted more than scientific output used in policy making. The antiscientific sentiment seems overlapping with the third group, but the source of distrust also differs.

Recent discussions on covid vaccines have underscored that trust is crucial in order to achieve high levels of immunisation (Verger and Dubé, 2020; Larson *et al.*, 2021), hence maintaining and building trust are important. Therefore, it makes sense to understand sources of distrust and see if these can be overcome. For example, if we find that health literacy of people on vaccines is low, we can use information strategies to help them make an informed choice. This is often how strategies to reach out to non-compliant groups in preventive medicine work (Ozawa *et al.*, 2019). What is used then, is a so-called ‘information deficit model’ where non-compliance is conceived in terms of people’s lack of knowledge, misinformation or a need for correcting irrational or biased choices (ALLEA, 2018). The deficit model assumes we can ‘fix’ the trust. However, the model has been criticised for being disrespectful, and to ignore the sources of distrust. De Melo-Martin states in a book review on Maya Goldenberg’s book *Vaccine Hesitancy*: ‘the publics are not ignorant, but distrustful’ (de Melo-Martín, 2021: 2). She also rejects the deficit model because it is one where vaccine advocates hold the moral ground and parents have to be convinced and turned (ibid). Instead, according to De Melo-Martin, Goldenberg sketches a story where trust is the result of multiple factors, including medical racism (ibid). Distrust has, in other words, also to do with being ignored, exploited or excluded as moral agent. This view finds support and is even more strongly claimed as a case of epistemic injustice by Cassam (2023). I will not discuss this epistemic claim further here in depth, but want to underscore the claim made by de Melo-Martín, that ‘attention to trust—or the lack thereof—when dealing with vaccine hesitancy and refusal is likely to be more productive than the traditional way of framing the problem’ (de Melo-Martín, 2021: 2).

How do Pierik and Verweij discuss trust in their book? After having laid out the sources of distrust in Chapter 3, they seem to share the criticism on the information-deficit model, when they state in Chapter 9 that it would be ‘disrespectful for governments to shape all information processes, communication policies and other social factors in a manner that maximises the public’s confidence in government policy and governmental actors. Information and communication then become not elements that enable people to make their *own* judgements and decisions, but elements that influence their perception and judgement in such a way that they will choose what the government prefers them to choose.’ (Pierik and Verweij, 2024: 176–7). This is a clear rejection of a ‘fixing-model’. They suggest to focus instead on *trustworthiness* and embrace a relational view on trust, where ‘we can see trust as a *mutual relationship* between (in this case) public health authorities (or government) and citizens’ (ibid:177). Governments should focus on building and maintaining relationships of trust and also ‘trust’ that if citizens have been offered appropriate information, they must be considered ‘as capable of making a good judgement about what to do’ (ibid).

I find an interesting addition to this perspective on trust which is not discussed by Pierik and Verweij, which is also part of a relational concept on trust. The authors in a paper on trust in science distinguish an affective component to trust that ‘revolve around questions of taking a chance in trusting someone, in putting ourselves at risk and demonstrating vulnerability’(ALLEA, 2018: 4). The authors of the Allea paper also reject the ‘deficit model’ and suggest that the affective component requires to take ‘the affective and social aspects of judgements of trustworthiness better into account’ (ibid).

A mutual relation of trust takes seriously the moral agency of parents and accounts for ‘the vulnerability that parents experience in conditions of perceived or real uncertainty’ (ibid). I think Pierik and Verweij might actually still embrace the information deficit model when they recommend later on in the chapter that ‘concerns of hesitant parents are taken seriously, but health authorities and professionals are also active in pointing out the flaws of misinformation or false beliefs’ (Pierik and Verweij, 2024: 186). Engagement requires, in my view, a more radical turn in the attitude towards the persons whose trust we seek. Maybe we need to look at it from a perspective of creating moral spaces (Walker, 2003) in order to address the affective component better. Taking the other serious is not only about presenting oneself as trustworthy, as Pierik and Verweij suggest, but requires being invested in the other, and fully understanding the sources of distrust. It is about supporting the other to decide if they want to take *the leap of faith* that is needed in trust. I think that Pierik and Verweij have done a great job in their book with regards to the arguments on coercive measures, but that the puzzle on trust remains: it requires on the one hand to fully understand the sources of distrust, while at the same time it should not encourage a ‘fixing attitude’ and if we embrace a relational view on trust, it will have more far-reaching consequences in our approach to non-compliant parents than Pierik and Verweij suggest. And if the sources of distrust are also related to epistemic injustices, as de Melo-Martín and Cassam point out, the more serious we need to rethink how governments can best bridge the distrust gap. To me, it remains an open question if discussions on justifications for coercive measures will bridge that gap.

## Notes

1
Trangerud (2023) does indicate that amongst religious objectors there are also persons who distrust vaccines, but this is only for a small group of religious objectors.
